# ZEB1 induces EPB41L5 in the cancer mesenchymal program that drives ARF6-based invasion, metastasis and drug resistance

**DOI:** 10.1038/oncsis.2016.60

**Published:** 2016-09-12

**Authors:** A Hashimoto, S Hashimoto, H Sugino, A Yoshikawa, Y Onodera, H Handa, T Oikawa, H Sabe

**Affiliations:** 1 Department of Molecular Biology, Hokkaido University Graduate School of Medicine, Sapporo, Japan

## Abstract

Onset of the cancer mesenchymal program is closely associated with cancer malignancy and drug resistance. Among the different epithelial–mesenchymal transition (EMT)-associated transcriptional factors, ZEB1 has a key role in inducing the mesenchymal phenotypes and stem cell-like properties of different breast cancer cells. ARF6 and its effector AMAP1 are frequently overexpressed in breast cancer cells, and promote invasion, metastasis and drug resistance. EPB41L5 is induced during EMT, and mediates the disruption of E-cadherin-based cell–cell adhesion and the promotion of focal adhesion dynamics. Here we show that EPB41L5 is an integral component of the ARF6-based pathway, which is induced by ZEB1. We found that EPB41L5 is expressed at high levels in malignant breast cancer cells and binds to AMAP1. ZEB1 induced *EPB41L5* both in cancer cells and normal cells. This relationship was recaptured with The Cancer Genome Atlas RNASeq data set, and correlated with the poor outcome of the patients. In contrast, diversified events, such as tumor growth factor β1 stimulation, expression of SNAI1 and *TP53* mutation, can each cause the induction of *ZEB1* and EPB41L5, depending on the cellular context. Our results demonstrated that the ZEB1-EPB41L5 axis is at the core of the cancer mesenchymal program that drives ARF6-based invasion, metastasis and drug resistance of significant populations of primary breast cancers, and is tightly correlated with the poor outcomes of patients.

## Introduction

The acquisition of malignant phenotypes by breast cancer cells often involves their transition into mesenchymal-type cells, through processes resembling epithelial–mesenchymal transition (EMT).^1, 2^ Such mesenchymal-type malignancy involves resistance against anoikis,^3, 4, 5^ which might help to maintain cell viability in the absence of cell adhesion during the distant metastasis of cancer cells, whereas mesenchymal malignancy on its own also includes highly invasive and metastatic potentials.^6^ Recent studies have moreover suggested that the acquisition of mesenchymal properties of cancer cells is closely related to drug resistance.^7, 8^

Research on breast cancer has played leading roles towards understanding the molecular mechanisms involved in cancerous EMT. High expression of certain transcriptional factors in breast cancer cells, which are induced during EMT (that is, EMT transcriptional factors), such as TWIST, SNAIL and ZEB, were found to be critical to the acquisition of invasive phenotypes to be correlated with the poor outcome of patients.^1, 2, 9, 10, 11^ ZEB1 has moreover been implicated in the cancer stem cell-like phenotypes.^12^ On the other hand, tumor growth factor (TGF)β1 signaling was found to be specifically upregulated in CD44^+^ cancer stem cell-like cells of many primary breast tumors, in which the robust presence of TGFβ1 signalsomes was highly coincident with the appearance of mesenchymal phenotypes.^13^ Consistently, TGFβ1 induces EMT of immortalized mammary epithelial cells *in vitro*, to be coupled with the generation of stem cell-like phenotypes.^14^ Mutation of the *TP53* gene, which encodes the tumor suppressor p53 protein, has also been shown to be closely related to the induction of EMT and the generation of cancer stem cell-like cells.^15, 16, 17^ However, proteins that are induced as a result of EMT and execute cancer mesenchymal malignancies still remain largely elusive.

The small-GTPase ARF6 is primarily involved in the recycling of plasma membrane components.^18^ ARF6 and its downstream effector AMAP1 (also called DDEF1 or ASAP1) are frequently overexpressed in different breast cancer cells and promote invasion, metastasis and drug resistance.^19, 20, 21, 22, 23^ In this pathway, ARF6 can be activated by GEP100 (also called BRAG2) under receptor tyrosine kinases, such as epidermal growth factor receptor.^24^ Mechanistically, the ARF6-based pathway disrupts E-cadherin-based adhesion^24^ and promotes recycling of β1 integrins;^25^ hence appears to drive EMT processes. Clinically, the robust expression of components of this pathway statistically correlates with the malignant phenotypes of human primary breast tumors, including rapid local recurrence after breast conservative therapy.^21, 24, 26^

EPB41L5 is a mesenchymal-specific protein induced during EMT of mammary epithelial cells.^27^ EPB41L5 binds to p120 catenin (p120) and hence sequesters p120 from E-cadherin, which causes internalization of E-cadherin.^27^ EPB41L5 also binds to the focal adhesion protein paxillin, and promotes focal adhesion dynamics, which likely enhances cell motility.^27^

Our previous studies indicated that breast cancer cells that bear mesenchymal properties use the ARF6-based pathway for invasion and metastasis.^20, 21, 24, 25, 28^ Here, we show that the ARF6-based pathway possesses a mesenchymal property, in which AMAP1 binds to EPB41L5; and that EPB41L5 is primarily induced by ZEB1 during the breast cancer mesenchymal program triggered by various events.

## Results

### EPB41L5 binds to AMAP1

**Figure 1:**
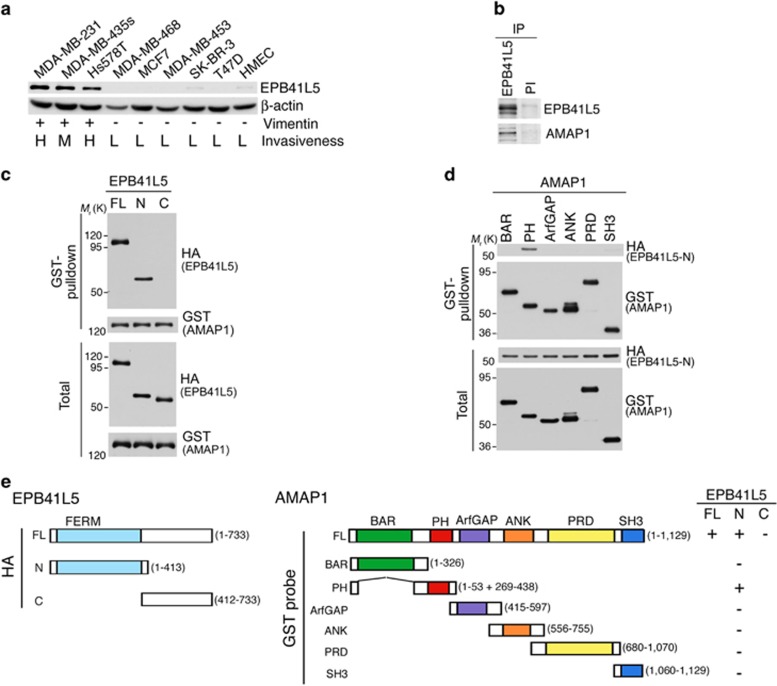
Expression of EPB41L5 in malignant breast cancer cells as an integral binding partner of AMAP1. (**a**) EPB41L5 expression among various breast cancer cell lines, detected by anti-EPB41L5 immunoblotting. β-actin was used as a control. Vimentin expression (+, −) and invasiveness (H, high; M, medium; L, low) are from the literature. (**b**) AMAP1 co-precipitated with an anti-EPB41L5 immunoprecipitation (IP) from MDA-MB-231 cell lysate was detected by its immunoblotting. Pre-immune serum (PI) was included as a control. (**c**–**e**) The FERM domain of EPB41L5 binds to the PH domain of AMAP1. Each domain of EPB41L5, as indicated in (**e**) and tagged with HA, was incubated with GST-AMAP1 bound to glutathione beads (**c**), or the HA-tagged N-terminal domain of EPB41L5 was incubated with each domain of GST-AMAP1, as indicated in (**e**) and bound to glutathione beads (**d**) to examine their binding. Proteins were expressed in HEK293 cells, and proteins co-precipitated with glutathione beads were analyzed by immunoblotting, as indicated. Total, total cell lysate (10 μg).

We first found that EPB41L5 is highly expressed in breast cancer cells, including MDA-MB-231, which exhibit mesenchymal properties (that is, are vimentin-positive^29^) and express ARF6 and AMAP1 at high levels^20, 21^ (Figure 1a). EPB41L5 binds to paxillin,^27^ which is an integral component of the invadopodia of breast cancer cells.^30^ AMAP1 is also an integral component of invadopodia and binds to paxillin.^21^ We found that AMAP1 is co-precipitated with anti-EPB41L5 from MDA-MB-231 cell lysates (Figure 1b). We then co-overexpressed full-length AMAP1 tagged with GST, and full-length EPB41L5 tagged with hemagglutinin (HA), in HEK293T cells, and confirmed their binding (Figure 1c). Deletion analyses revealed that the PH domain of AMAP1 and the N-terminal half of EPB41L5 (mostly consisting of the 4.1 protein, ezrin, radixin and moesin (FERM) domain) primarily mediate this binding (Figures 1c–e).

### EPB41L5 is crucial for invasion and metastasis

**Figure 2:**
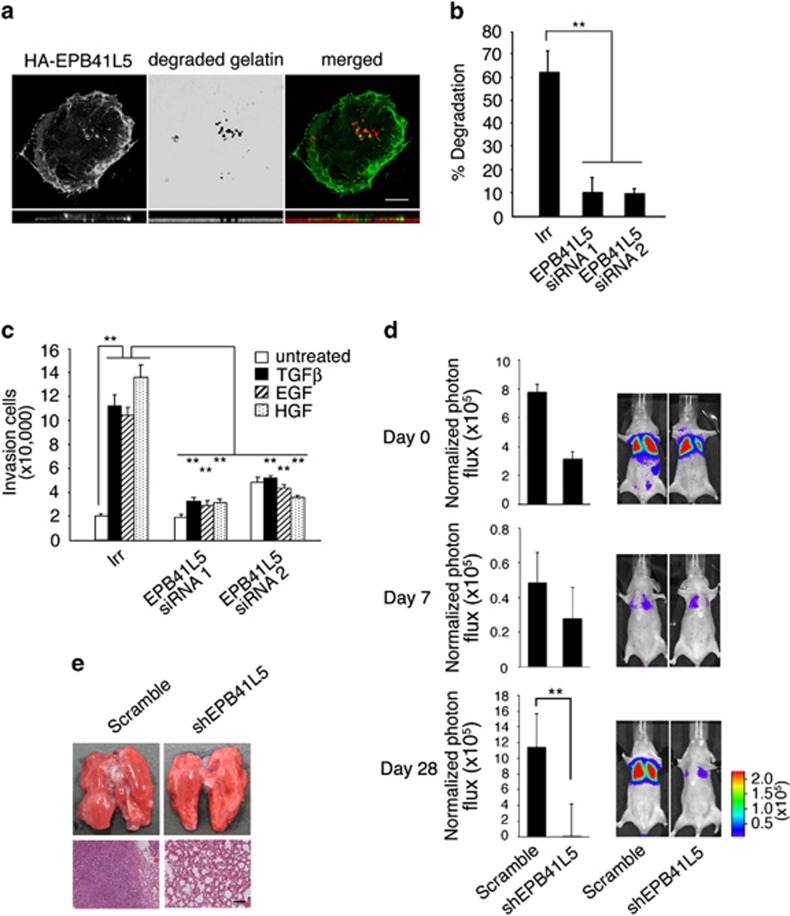
Requirement for EPB41L5 in invadopodia formation, invasion and metastasis. (**a**) Accumulation of EPB41L5 at invadopodia. MDA-MB-231 cells expressing HA-EPB41L5 were cultured on a gelatin matrix. Confocal images of cells stained with an anti-HA antibody (left panel, and green in right panel), areas of degraded gelatin (middle panel, and red in right panel), and their merged image (right) are shown. *Z*-axis images are shown below. Scale bar, 10 μm. (**b** and **c**) Requirement for EPB41L5 in the matrix degradation (**b**) and invasion (**c**). Percentage of cells exhibiting the gelatin degradation (**b**) and the ligand-induced Matrigel invasion activities (**c**) were measured using MDA-MB-231 cells pre-treated with siRNAs for *EPB41L5* (#1 and #2) or with an oligonucleotide bearing an irrelevant sequence (Irr). All data represent the average of three independent experiments in duplicates. The results represent means±s.e.m. **P<0.01. (**d** and **e**) Requirement for EPB41L5 in metastasis. MDA-MB-231 cells, expressing a luciferase reporter and transfected with an shRNA construct to silence *EPB41L5* or with a control vector (Scramble), were injected into tail veins of nude mice. (**d**) Bioluminescence intensities of the chests of injected mice were measured on indicated days after injection. The results are shown as means±s.e.m. (*n*=5 for each). **P<0.01. Representative whole images of mice are shown on the right. (**e**) Representative whole images of lungs (top) and H&E staining of the sections (bottom) on the 28 days post injection. Scale bar, 100 μm.

Similarly to ARF6 and AMAP1,^20, 21^ HA-tagged EPB41L5 was found to accumulate at the invadopodia of MDA-MB-231 cells, which were formed upon the degradation of collagen matrices (Figure 2a). Small interfering RNA (siRNA)-mediated silencing of *EPB41L5* blocked invadopodia formation of MDA-MB-231 cells, as well as their invasion through the Matrigel barrier (Figures 2b and c, Supplementary Figures S1a and b). Silencing of *EPB41L5* also blocked the lung metastasis of MDA-MB-231 cells in nude mice, in which cells were originally injected into tail veins (Figures 2d and e, Supplementary Figures S1c–e). We confirmed that this silencing did not notably affect cell growth *in vitro* (Supplementary Figure S1f). Together with the above results, our results indicated that EPB41L5 is an integral binding partner of AMAP1 to promote invasive and metastatic activities of breast cancer cells.

### ZEB1 induces *EPB41L5* in breast cancer

**Figure 3:**
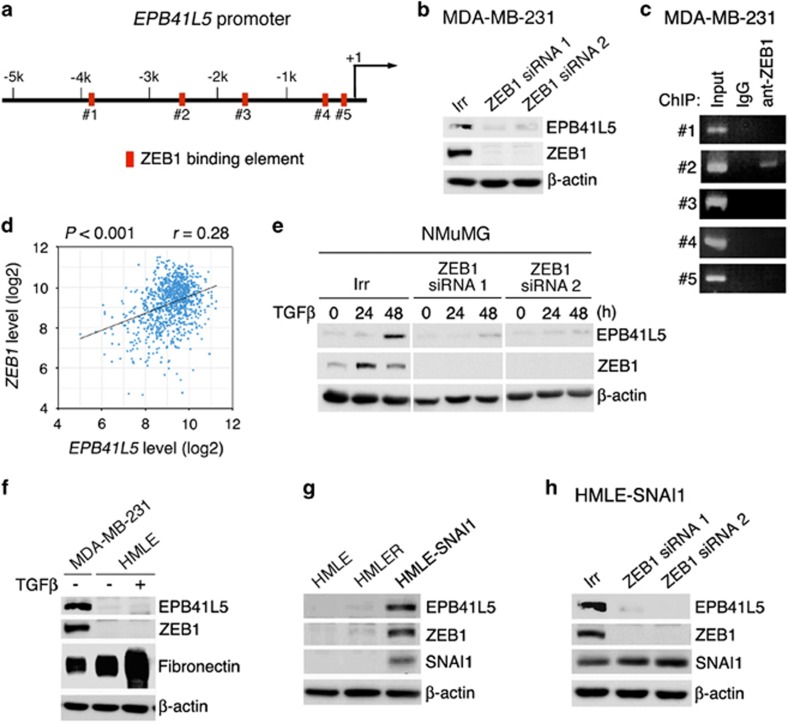
ZEB1-EPB41L5 axis in transformed and non-transformed mammary epithelial cells. (**a**) Putative ZEB1-binding sites within the *EPB41L5* gene promoter. (**b**) ZEB1 is responsible for EPB41L5 expression in MDA-MB-231 cells. Cells were treated with siRNAs for *ZEB1* or Irr. (**c**) A ChIP assay of ZEB1-binding to the *EPB41L5* promoter using antibodies against ZEB1 or a control IgG. (**d**) Statistic correlation of the *ZEB1* mRNA levels with the *EPB41L5* mRNA levels in TCGA RNASeq data set on human primary breast tumors (*n*=970). A *P*-value represents the results of the Spearman rank correlation test. (**e**) ZEB1 is responsible for the TGFβ1-induced EPB41L5 expression in NMuMG cells. Cells were pre-treated with siRNAs for ZEB1 (#1 and #2) or Irr, before being incubated with TGFβ1 for the indicated times. (**f**) TGFβ1 does not induce ZEB1 or EPB1L5 in HMLE cells. Cells were incubated with TGFβ1 for 12 d. (**g**) SNAI1, but not oncogenic Ras signaling, induces ZEB1 and EPB41L5 in HMLE cells. (**h**) ZEB1 is responsible for EPB41L5 expression in HMLE-SNAI1 cells. Cells were treated with siRNAs for *ZEB1* or Irr. In (**b**) and (**e**–**h**), protein expression was analyzed by immunoblotting by using antibodies, as indicated. A control included a β-actin immunoblotting.

We next sought to identify EMT transcriptional factors primarily responsible to induce *EPB41L5* in breast cancer cells. The *EPB41L5* gene promoter contains putative binding sites for several EMT transcriptional factors, including ZEB1 (Jaspar [http://jaspar.genereg.net/] and MatInspector software,^31^ Figure 3a, Supplementary Figure S2a). MDA-MB-231 cells expressed ZEB1, and silencing of *ZEB1* almost completely abolished EPB41L5 expression, both at the mRNA and protein levels (Figure 3b, Supplementary Figure S2b). Among the five putative ZEB1-binding sites of the *EPB41L5* gene promoter, binding of ZEB1 with site #2 was clearly detected in MDA-MB-231 cell lysates, as assessed by the chromatin immunoprecipitation assay using an anti-ZEB1 antibody (Figure 3c, Supplementary Figure S2a). Consistently, The Cancer Genome Atlas RNASeq data set on human primary breast tumors (*n*=970) indicated that high levels of *ZEB1* mRNA statistically correlate with high levels of *EPB41L5* mRNA (Figure 3d). Therefore, ZEB1 is likely to have a key role in induction of the *EPB41L5* gene in significant populations of primary breast tumors.

### ZEB1 induces *EPB41L5* also in non-transformed cells

To assess whether the induction of *EPB41L5* by ZEB1 is a general event, we next investigated whether this induction occur also in non-transformed mammary epithelial cells. The NMuMG cell line is derived from mouse normal mammary epithelial cells,^32^ in which TGFβ1 induces EPB41L5 at the mRNA and protein levels.^27^ We found that TGFβ1 induces ZEB1 expression in these cells, whereas silencing of *ZEB1* almost completely abolishes TGFβ1-induced EPB41L5 expression (Figure 3e). HMLE cells are immortalized human mammary epithelial cells.^33^ HMLE cells expressed only marginal levels of EPB41L5 and ZEB1 (Figure 3f). TGFβ1 did not notably enhance the expression of ZEB1 and EPB41L5 in HMLE cells, whereas this stimulation promoted the expression of fibronectin, which is indicative of progression of some EMT process (Figure 3f, Supplementary Figure S2c). On the other hand, the transcriptional factor SNAI1 was reported to induce ZEB1^34^. ZEB1 and EPB41L5 were clearly expressed in HMLE cells in response to exogenously expressed SNAI1 (Figure 3g, Supplementary Figure S2c), in which silencing of *ZEB1* almost completely abolished SNAI1-induced EPB41L5 expression (Figure 3h). HMLER cells were derived from HMLE cells by transfection with V12H-RAS.^33^ *ZEB1* and *EPB41L5* were not expressed in HMLER cells (Figure 3g). Therefore, these results support a notion that the ZEB1-EPB41L5 axis exists also in non-transformed cells. Our results also indicated that TGFβ1 might not be a general inducer of the ZEB1-EPB41L5 axis, and oncogenic RAS may also not be a common inducer of this axis.

### *TP53* mutation induces *ZEB1* and *EPB41L5*

**Figure 4:**
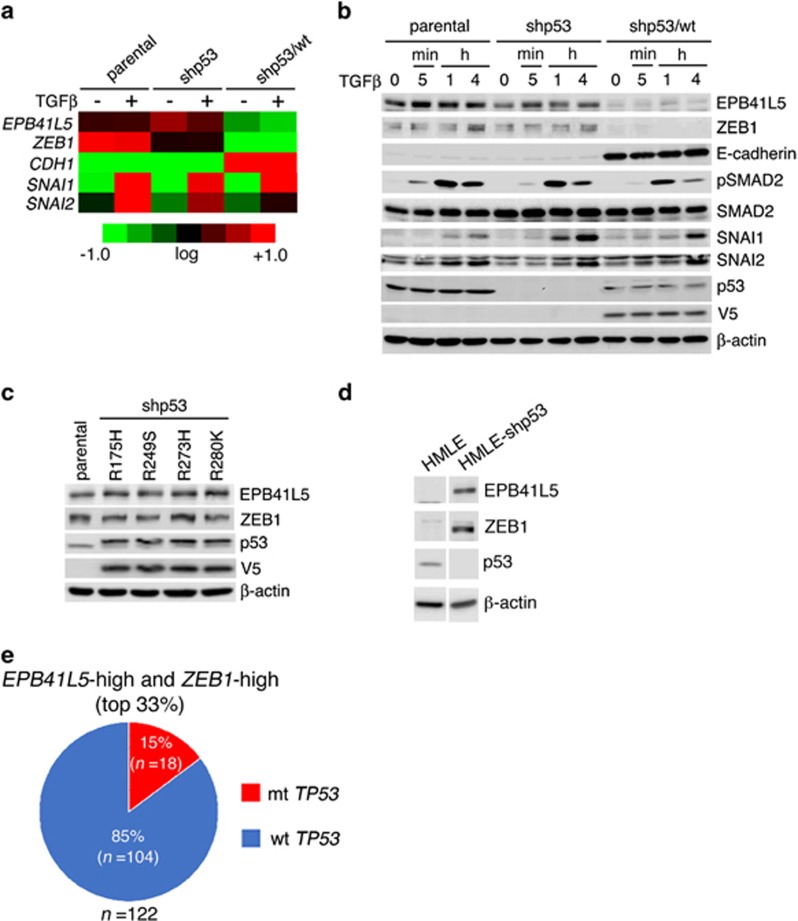
Loss of normal-p53 induces ZEB1 and EPB41L5. (**a**–**c**) Altered expression of ZEB1 and EPB41L5, and the other EMT-related genes (**a**) and their protein products (**b**) in MDA-MB-231 cells (parental) and their p53 derivatives, stimulated with or without TGFβ1 for 2 h (**a**), or as indicated (**b**). (**c**) Restoration of the EPB41L5 expression in shp53 cells by the rescue construct of R175H, R249S, R273H and R280K. In (**a**), mRNA levels below, equal to, or above the mean are indicated in green, black and red, respectively, in which the expression intensities are represented in the log2 scale. (**d**) Induction of ZEB1 and EPB41L5 in HMLE cells upon silencing *TP53* (HMLE-shp53). In (**b**–**d**) cell lysates (10 μg each) were analyzed by immunoblotting, as indicated. A control included a β-actin immunoblotting. (**e**) Distribution of *TP53* status in the simultaneous high expression of *EPB41L5 and ZEB1* mRNAs of breast cancers (*n*=122).

Normal-p53 has a potential to suppress the mesenchymal program of breast cancer.^15, 16^ Mutations in the *TP53* gene are frequent in breast cancer.^35^ We next investigated the possible involvement of *TP53* mutations in induction of the ZEB1-EPB41L5 axis. MDA-MB-231 cells express an oncogenic mutant-p53, R280K, together with loss of the other *TP53* allele.^36^ We generated MDA-MB-231 cells in which endogenous mutant-p53 was silenced (shp53 cells), as has been done previously.^37^ We moreover generated shp53 cells expressing V5-tagged normal-p53 (shp53/wt cells), in which expression levels of normal-p53 were carefully tuned to avoid cell senescence and death (Supplementary Figure S3a; also see Materials and methods). Shp53 cells expressing the rescue construct (shp53/R280K cells), or expressing the other p53-mutants, R175H, R249S and R273H, which were all thought to be oncogenic, were also generated. Expression of ZEB1 and EPB41L5, at the mRNA and protein levels, was found to be repressed in shp53/wt cells, whereas they were expressed in shp53 cells and also in all of these cells expressing p53-mutants, at levels similar to the parental cells (Figures 4a–c, Supplementary Figure S3b). On the other hand, TGFβ1 did not affect the expression of ZEB1 and EPB41L5 in any of these cells, whereas TGFβ1 induced SNAI1 and SNAI2 in these cells, irrespective of the *TP53* status (Figures 4a and b). Expression of *TWIST1/2* mRNA was almost undetectable (Supplementary Figure S3c). Therefore, loss of normal-p53 function appeared to be an event that induces the ZEB1-EPB41L5 axis in MDA-MB-231 cells. On the other hand, our results revealed that SNAI1 can induce *ZEB1* in a cell-context-dependent manner.

The induction of ZEB1 and EPB41L5 upon the silencing *TP53* was observed in HMLE cells (Figure 4d), whereas we have also observed that the silencing of *TP53* does not induce EMT-associated processes in another preparation of HMLE cells (data not shown). Using The Cancer Genome Atlas RNASeq data set, we then sought to assess the extent to which *TP53* mutations are associated with induction of the ZEB1-EPB41L5 axis in primary breast tumors. We classified the top 33% of primary breast tumors regarding their high expression of both *ZEB1* mRNA and *EPB41L5* mRNA as the high-expression group, and found that ~15% of tumors of the high-expression group bear *TP53* mutations (Figure 4e). Therefore, although the high expression of *ZEB1* and *EPB41L5* appears to occur among certain populations of primary breast tumors bearing *TP53* mutations, these results suggested that the loss of normal-p53 might not be a major cause inducing the ZEB1-EPB41L5 axis and that induction of this axis may frequently occur even in the presence of normal-p53.

### The ARF6-AMAP1-EPB41L5 pathway promotes drug resistance

We have already shown that EPB41L5 promotes the drug resistance of breast cancer cells, such as MDA-MB-231 and MDA-MB-435s, both of which express every component of the ARF6-based pathway at high levels.^28^ Involvement of ZEB1 in drug resistance has also been documented in pancreatic cancers.^38, 39, 40^ We then sought to understand whether other components of the ARF6-based mesenchymal pathway are also involved in promoting the drug resistance. Gemcitabine is a cytidine analog, and fluorouracil is a pyrimidine analog.^41^ Temsirolimus is an inhibitor of mTOR activity.^42^ Similar to the silencing of *EPB41L5*, siRNA-mediated silencing of *ZEB1* and *AMAP1* in MDA-MB-231 cells and MDA-MB-435s cells resulted in reduced cell survival upon treatment with Gemcitabine, fluorouracil or Temsirolimus, compared with the parental cells treated with control siRNAs (Supplementary Figures S4a–i). On the other hand, silencing of ARF6 for more than 2 days affected the growth of these cells, and thus we were unable to precisely assess the effects of ARF6 silencing on drug resistance. Collectively, it is conceivable that rather than the solitary expression of ZEB1 or EPB41L5 at high levels, high expression of the intact ARF6-based mesenchymal pathway is necessary to render the drug resistance. Moreover, our results also suggest that induction of EPB41L5 is critical for the ZEB1-mediated drug resistance.

### High expression of *EPB41L5* statistically correlates with poor outcome of patients

**Figure 5:**
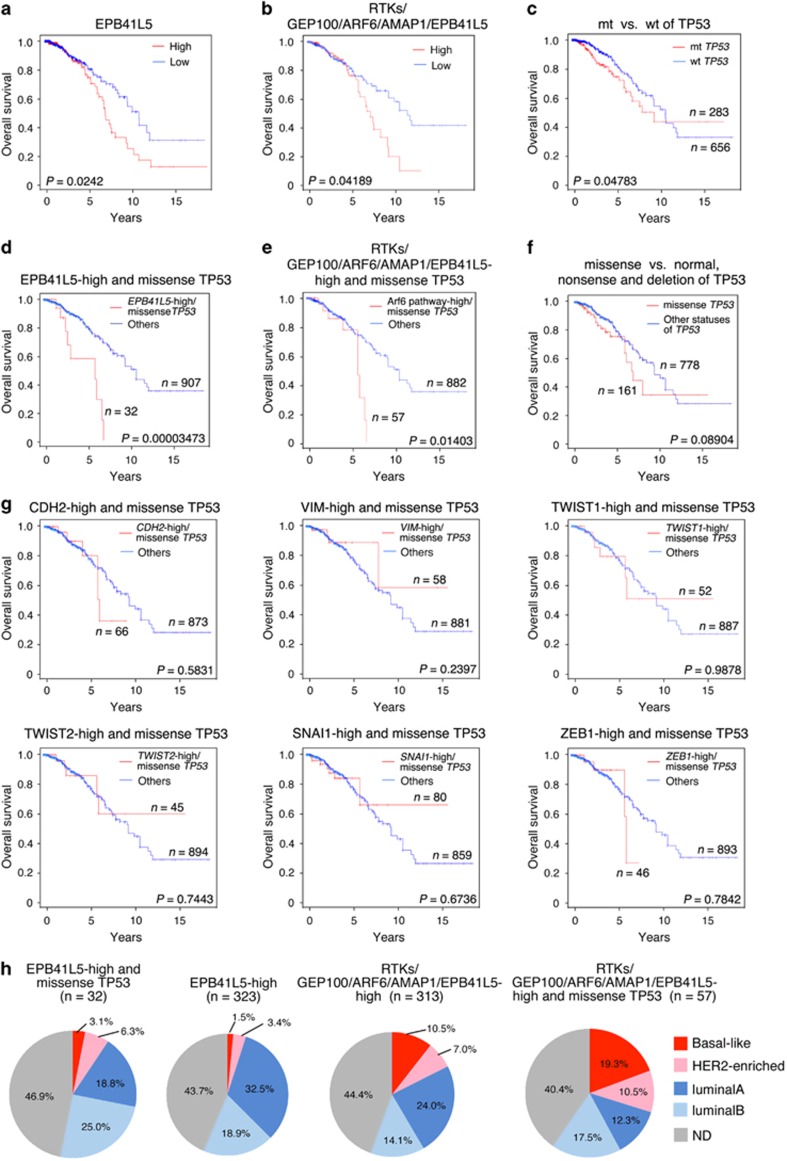
EPB41L5 as a biomarker for poor outcome of breast cancer patients. (**a**–**f**) TCGA RNASeq database on primary breast tumors (*n*=970) was analyzed with regard to the high levels of *EPB41L5* (**a**), the simultaneous high expression of mRNAs encoding all of the ARF6 pathway components (**b**), the *TP53* status (mutants (mt) vs normal (wt)) (**c**), co-incidence of the *EPB41L5-*high expression with the *TP53* missense mutations (**d**), co-incidence of the simultaneous high expression of all of the ARF6 pathway mRNAs with the *TP53* missense mutations (**e**), and the *TP53* missense mutations alone (**f**). (**g**) Co-incidence of the *TP53* mutations with high expression of other EMT-related genes, as indicated, does not correlate with patient survival. The database was analyzed by including the top 33% of patients regarding the expression levels of the indicated mRNAs in the high-expression group. (**a**–**g**) Survival rates of each group are shown as Kaplan–Meier curves, and *P*-values represent the results of the log-rank test. (**h**) Distribution of the four main molecular classes of breast cancers in the *EPB41L5*-high together with the missense *TP53*, the *EPB41L5*-high, the ARF6 pathway-high (*RTKs/GEP100/ARF6/AMAP1/EPB41L5*-high), and the ARF6 pathway-high together with the missense *TP53*. ND, not determined.

By analyzing The Cancer Genome Atlas RNASeq data set on primary breast tumors, we then found that high expression (top 33%) of *EPB41L5* mRNA statistically correlated with the poor overall survival of patients (Figure 5a). Simultaneous high expression (top 33%) of every mRNA encoding a component of the ARF6-based pathway, namely, receptor tyrosine kinases (epidermal growth factor receptor and/or c-Met), GEP100, ARF6, AMAP1 and EPB41L5, also exhibited a statistical correlation with poor overall survival (Figure 5b).

It has been shown that *TP53* status on its own might not tightly correlate with the poor outcome of patients.^43^ This notion was recaptured in this The Cancer Genome Atlas data set with regard to the overall survival of patients (see Figure 5c). A significant population of *TP53* missense mutations may produce oncogenic mutant-p53 proteins.^17^ We found that the high expression of *EPB41L5* in the presence of *TP53* missense mutations tightly correlates with the short-term survival of patients, with a lower *P*-value than that of the high *EPB41L5* expression alone (Figure 5d). The same was true in the case of the high expression of all components of the ARF6 pathway together with *TP53* missense mutations (Figure 5e). On the other hand, *TP53* missense mutations alone did not correlate with poor survival (Figure 5f). *CDH2* (encoding N-cadherin), *VIM* (encoding vimentin), *TWIST1/2* (products which are EMT transcriptional factors) and *SNAI1,* as well as *ZEB1*, are well known key players involved in EMT-like processes of breast cancers, as mentioned earlier. The high expression of any of these mRNAs, by their own or even in the presence of missense *TP53* mutations, also did not statistically correlate with the poor survival of patients (Figure 5g, Supplementary Figure S5a). These results suggested that a high expression of *EPB41L5* play a central role in the poor outcome of breast cancer patients, rather than high expression of the other EMT-related genes, including *ZEB1*. In this regard, it should be noted that the *EPB41L5* gene is not the sole target of ZEB1. Our analysis moreover suggested that high levels of *EPB41L5* expression may become more closely associated with the poor overall survival of patients, if cells possess oncogenic mutations of *TP53* (see later).

### High expression of *EPB41L5* is not specifically associated with basal-like or HER2-enriched genotypes

Primary breast cancers can be categorized into four main molecular classes regarding their gene expression profiles: basal-like, HER2-enriched, luminal A and luminal B.^35^ *TP53* mutations are very frequent in the basal-like (80%) and HER2-enriched (72%) genotypes, which exhibit very aggressive phenotypes compared with the luminal A/B types. We found that high expression of *EPB41L5* is not specifically related to the basal-like or HER2-enriched genotypes, but appears to be rather related to the luminal A/B genotypes (Figure 5h, Supplementary Figure S5b). High expression of *EPB41L5* in the presence of *TP53* missense mutations was not specifically related to any of the four genotypes (Figure 5h, Supplementary Figure S5b). High mRNA expression of all components of the ARF6-based pathway, as well as their co-incidence with *TP53* missense mutations, also appeared to occur almost randomly among these four molecular classes (Figure 5h, Supplementary Figure S5b). Therefore, it is likely that EBP41L5-mediated mesenchymal phenotypes may arise mostly independent of the basal-like and HER2-enriched genotypes. This notion is consistent with the above results that *TP53* mutations might not be the major cause inducing *EBP41L5* among different breast cancers.

## Discussion

In this study, we show that the ARF6-based pathway includes a mesenchymal-specific protein, EPB41L5, as its integral component. Therefore, this pathway appears to be a mesenchymal-specific pathway, not expressed in cancer cells unless they undergo EMT. We have moreover demonstrated that this pathway contributes to the drug resistance, rather than merely promoting invasion and metastasis. There is an argument regarding whether EMT-like changes of cancer cells actually contribute to their invasion and metastasis, whereas such changes might contribute to the drug resistance.^44, 45, 46^ Our results, however, clearly demonstrated that the ARF6-AMAP1-EPB41L5 mesenchymal pathway is critical to both the invasion/metastasis and the drug resistance. We are studying precise molecular mechanisms as to how this pathway contributes to the drug resistance.

ZEB1 was identified to be primarily responsible to induce *EPB41L5* in both non-transformed and transformed mammary epithelial cells, whereas several different events, such as TGFβ1, SNAI1 and *TP53* mutation, induced the ZEB1-EPB41L5 axis. TGFβ1 has been well documented to be associated with mesenchymal properties by breast cancers.^13^ However, it is rare that this cytokine alone is sufficient to induce EMT.^47, 48^ Our results indicated that the induction of the ZEB1-EPB41L5 axis by TGFβ1 does not always occur among different mammary epithelial cells. Likewise, although the expression of SNAI1 has been shown to induce *ZEB1*^34^ and to be critical to the malignancy of certain populations of primary breast cancers,^49^ SNAI1 did not necessarily induce *ZEB1*. Furthermore, although we observed that loss of normal-p53 induces the ZEB1-EPB41L5 axis in MDA-MB-231 cells, loss of normal-p53 does not always induce the ZEB1-EPB41L5 axis among different preparations of HMLE cells. Clinical data set analysis indicated that *TP53* mutation might not be the major cause that induces high expression of *ZEB1* and *EPB41L5* among different primary breast tumors. Therefore, whereas the ZEB1-EPB41L5 axis appears to be central to promote mesenchymal malignancies of significant populations of primary breast cancers, different events may initially trigger induction of this axis perhaps dependent on cell contexts. It is well documented that epigenomic chromatin modifications are critical in determining the *ZEB1* transcription,^12^ and that miRNAs are involved in regulating the *ZEB1* transcripts.^50^ We have yet to understand what types of genome/epigenome statuses of cancer cells, as well as the microenvironments, are involved in the apparently non-linear processes inducing the ZEB1-EPB41L5 axis. Furthermore, although ZEB1 can be critical in the cell plasticity generating cancer stem cells,^12^ we have yet to study the relationship between EPB41L5 expression and the generation of cancer stem-like cells.

The high expression of *EPB41L5* in the presence of *TP53* missense mutations was more tightly correlated with the poor overall survival of patients than the high expression of *EPB41L5* alone. We have shown that upregulation of the mevalonate pathway by oncogenic mutant-p53^51^ is critical to facilitate ARF6 activation.^28^ On the other hand, *TP53* mutations are not the major event inducing the ZEB1-EPB41L5 axis. Therefore, these two events, induction of the ZEB1-EPB41L5 axis and *TP53* mutations, can be complementary to each other in generating and activating the ARF6-based mesenchymal pathway, and may hence cooperatively promotes the mesenchymal malignancy of breast cancer cells.

In conclusion, our study demonstrated that the ZEB1-EPB41L5 axis is at the core of the mesenchymal program of breast cancer, which carries out ARF6-mediated invasion, metastasis and drug resistance. Our analyses also demonstrated that a high expression level of *EPB41L5* is a key factor in the poor overall survival of patients, rather than the high expression level of other representative genes associated with EMT. Collectively, the ARF6-based pathway driven by the ZEB1-EPB41L5 axis appears to account for almost half the population of breast cancer patients that survive for less than several years after diagnosis under current therapeutics.

## Materials and methods

### Cells

MDA-MB-231, MDA-MB-435s, Hs578T, NMuMG and human mammary epithelial cells were obtained from the American Type Culture Collection (ATCC, Manassas, VA, USA). MDA-MB-231 cells were maintained in a 1:1 mixture of DMEM (Sigma-Aldrich, St Louis, MO, USA) and RPMI 1640 (Sigma-Aldrich) supplemented with 10% fetal calf serum (FCS, HyClone, Thermo Scientific, Logan, UT, USA) and 5% NU serum (BD Biosciences, San Jose, CA, USA), as described previously.^20^ MDA-MB-435s, Hs578T, NMuMG and human mammary epithelial cells were maintained as instructed by the ATCC. HEK293T cells were purchased from Invitrogen (Carlsbad, CA, USA), and maintained according to the manufacturer's instructions. Plat-E cells were a gift from Dr Kitamura (Tokyo University, Tokyo, Japan) and maintained in DMEM containing 10% FCS. HMLE and HMLER cells were gifts from Dr Weinberg (Whitehead Institute, MIT, Cambridge, MA, USA) and cultured in MEGM medium (Lonza, Walkersville, MD, USA). HMLE cells expressing Snail were generated via retroviral infection of Snail into HMLE cells (HMLE-Snail) and cultured in MEGM medium. No antibiotics were used in our cell cultures. All cell lines were routinely checked for mycoplasma contamination by propidium iodide staining as well as by the treatment with MC-210 (DS Pharma Biomedical, Osaka, Japan).

For ligand stimulation, cells were pre-starved for FCS for 16 h, and then incubated with TGFβ1 (2 ng/ml; R&D Systems, Minneapolis, MN, USA) in the absence of FCS for indicated times, before being subjected to analyses.

Cell viabilities were measured using a Cell counting kit-8 (CCK-8) (Dojindo, Kumamoto, Japan), according to the manufacturer's instructions. The experiments were repeated at least three times.

Cell cycle analysis was performed with a BrdU Flow Kit (BD Biosciences), according to the manufacturer's instructions using a FACS Canto II Cytometer (BD Biosciences).

### Plasmids

*AMAP1* complementary DNAs (cDNAs), encoding the full-length, the BAR domain (aa 1–326), the PH domain (aa 1–53, 269–438), the ArfGAP domain (aa 415–597), the ANK domain (aa 556–755), the PRD domain (aa 680–1070) and the SH3 domain (aa 1060–1129) were synthesized from the *AMAP1* cDNA^21^ using the PCR, and ligated into the *BamH1/NotI* site of pEBG^52^ to be fused to the C-terminus of GST. *EPB41L5* cDNAs, encoding the full-length, the N-terminal half (aa 1–413) and the C-terminal half (aa 414–733), were synthesized by use of mRNAs prepared from MDA-MB-231 cells, and ligated into the *BamH1/NotI* site of pcDNA3-HA (Invitrogen) to be fused to the C-terminus of the HA-tag. Oligonucleotides used for the PCR reactions are shown in Supplementary Table S2.

### Antibodies and immunoblotting

Affinity-purified rabbit polyclonal antibody against AMAP1 was as described previously.^21^ Rabbit polyclonal antibody against EPB41L5 was raised against a GST-fused peptide corresponding to amino acids (aa) 541–733 of EPB41L5. The resulting serum was adsorbed with GST and then affinity-purified using the antigen peptide, before use. Other antibodies were purchased from commercial sources: mouse monoclonal antibodies against p53 (#2524, Cell Signaling Technology, Beverley, CA, USA), V5-tag (#R960-25, Invitrogen), HA-tag (#MMS-101R, Biolegend, San Diego, CA, USA), E-cadherin (#610182, BD Biosciences), and β-actin (#A5441, Sigma-Aldrich); and rabbit polyclonal antibodies against Smad2 (#3103), Ser465/467-phosphorylated Smad2 (#3101), ZEB1 (#3396), Snail (#3879), and Slug (#9585) (all from Cell Signaling Technology). Donkey antibodies against rabbit (#711-036-152) and mouse immunoglobulins G (#711-036-151), each conjugated with horseradish peroxidase, were from Jackson ImmunoResearch Laboratories (West Grove, PA, USA).

Immunoblotting analysis was performed by use of ECL kit (GE Healthcare Life Sciences, Piscataway, NJ, USA), as described previously.^20, 21, 24, 25^

### Protein co-precipitation

Protein co-precipitation assays were performed as described previously.^24^ In brief, cells were lysed in RIPA buffer (1% Nonidet P-40, 1% deoxycholate, 0.1% sodium dodecyl sulfate, 20 mM Tris-HCl (pH 7.4), 150 mM NaCl, 5 mM ethylenediaminetetraacetic acid, 1 mM Na_3_VO_4_, 1 mM phenylmethylsulfonyl fluoride, 5 μg/ml aprotinin, 2 μg/ml leupeptin and 3 μg/ml pepstatin A). After being clarified by centrifugation, cell lysates (1 mg each) were incubated with antibodies coupled with Protein A-Sepharose beads. After being incubated for 1 h at 4 °C, proteins precipitated with beads were separated on sodium dodecyl sulfate-polyacrylamide gel electrophoresis (8% gel) and analyzed by immunoblotting.

### *In vitro* protein binding

*In vitro* protein binding assay was performed as described previously.^24^ In brief, 5 × 10^5^ HEK293T cells were transfected with 3 μg pEBG plasmid encoding GST-AMAP1 constructs and 0.3 μg of pcDNA3 plasmid encoding HA-EPB41L5 constructs, using Polyfect (Qiagen, Valencia, CA, USA). After incubating for 26 h, cells were lysed with NP-40 buffer (1% Nonidet P-40, 150 mM NaCl, 20 mM Tris-HCl pH 7.4, 5 mM ethylenediaminetetraacetic acid, 1 mM Na_3_VO_4_, 1 mM phenylmethylsulfonyl fluoride, 5 μg/ml aprotinin, 2 μg/ml leupeptin and 3 μg/ml pepstatin A). After being clarified by centrifugation, cell lysates (300 μg each) were incubated with 10 μl glutathione beads for 1 h at 4 °C. After washing, proteins bound to the beads were analyzed as above.

### siRNAs

For transient siRNA-mediated gene silencing, cells were transfected with 50 nM siRNA oligonucleotide duplexes using Lipofectamine 2000 or Lipofectamine RNAi Max (Invitrogen), according to the manufacturer's instructions. Duplex oligonucleotides were chemically synthesized and purified by Japan BioService. Two different sequences were used for each target, unless otherwise described. For silencing of human ZEB1 and mouse ZEB1, the ZEB1 stealth siRNAs (ZEB1-HSS110548, ZEB1-HSS110549, Zeb1-MSS210695 and Zeb1-MSS2106956; Invitrogen) were used, together with a negative control Stealth RNAi duplexes with medium GC content (Invitrogen). Nucleotide sequences used are shown in Supplementary Table S1.

### Invadopodia formation

Invadopodia formation assays were performed as described previously.^20, 21^ In brief, cells were transfected with the *EPB41L5*-specific siRNA oligonucleotides (sequence #1 or #2). After 48 h, cells were re-plated onto a culture dish coated with Alexa 594-labeled gelatin film, and cultured for an additional 16 h. Cells were then fixed in 4% paraformaldehyde in phosphate-buffered saline (PBS), and labeled using the appropriate antibodies and 4′,6-diamidino-2-phenylindole. The number of cells degrading the gelatin film was counted using a confocal laser-scanning microscope (Model A1R, Nikon, Tokyo, Japan).

For analysis of the subcellular localization of EPB41L5, cells were transfected with the pcDNA3-3HA-EPB41L5 plasmid and incubated for 24 h. Cells were then re-plated onto a culture dish coated with Alexa 594-labeled gelatin film, and cultured for an additional 8 h. After fixing in 4% paraformaldehyde in PBS, cells were labeled with an anti-HA antibody, and examined as above. Results from at least three independent experiments are shown.

### Metastasis assay

Nu/Nu athymic mice were obtained from CLEA Japan (Tokyo, Japan). All experiments were conducted under a protocol approved by the animal care committee of Hokkaido University. MDA-MB-231 cells were lentivirally infected with pLenti CMV V5-Luc blast (cat# 21474, Addgene, Cambridge, MA, USA), and a pLKO.1puro shRNA plasmid construct to silence *EPB41L5* or a control vector (a scrambled shRNA cat# 1864, Addgene). According to the past experience, a total of 2 × 10^6^ of these cells were injected into the lateral tail vein of each female mouse at the age of 5 weeks (*n*=5/group).

For bioluminescence imaging, mice were anaesthetized with 3% isoflurane and given 150 mg/kg D-luciferin in PBS by intraperitoneal injection. At 10 min after injection, bioluminescence was detected with an IVIS imaging system (Xenogen Corporation, Hopkinton, MA, USA) and analyzed with Living Image software (Xenogen Corporation). Photon flux (photons s/sr/cm) was calculated for each mouse using a region of interest encompassing the thorax. This value was normalized with a comparable background value. For histology, lungs were fixed in 10% neutral-buffered formalin (Wako, Tokyo, Japan). Sections were stained with hematoxilin using standard procedures by Morpho Technology (Hokkaido, Japan). The experiments were performed and analyzed in non-randomized and non-blinded fashion.

### Chromatin immunoprecipitation assay

Chromatin immunoprecipitations were performed by using the ChIP-IT Express Kit (Active Motif, Carlsbad, CA, USA), according to the manufacturer's instructions. In brief, MDA-MB-231 cells cultured on dishes were fixed in 1% formaldehyde for 5 min at room temperature, which was followed by incubation with 125 mM glycine in PBS. Cells were then collected by scraping, and their nuclei were extracted by passing through a 1 ml syringe with a 27 G needle 50 times and collected by centrifugation at 2400 *g* for 10 min at 4 °C. After being mixed with Enzymatic Shearing Cocktail for 10 min at 37 °C, nuclear lysates were incubated with 5 μg of an anti-ZEB1 antibody (sc-25388, Santa Cruz, Biotechnology, Santa Cruz, CA, USA) or control immunoglobulins G for 4 h at 4 °C, together with protein-G magnetic-beads. The beads were then washed four times, and subjected to elution. The eluents were then incubated with proteinase K overnight for 1 h at 37 °C, and subjected to DNA extraction to amplify the DNA fragments by RT-PCR. Primers for the PCR are shown in Supplementary Table S2.

### p53 manipulation and ectopic expression of SNAI1

For stable silencing of the endogenous mutant-p53 of MDA-MB-231 cells or the endogenous normal-p53 of HMLE cells, pLKO.1-Puro vector-based recombinant lentiviruses were generated, according to the method described previously.^53^ For generation of shp53 cells stably expressing normal- or mutant-p53 proteins, pBabe-based retroviruses^54^ were generated. cDNAs encoding V5-tagged normal-p53 protein and mutant-p53 proteins (R175H, R249S, R273H and R280K) were purchased from Addgene (cat# 22945, cat# 22936, cat# 22935, cat# 22934, and cat# 22933, respectively). Each of these DNA fragments was ligated into the *SnaB1* site of pBabe-Hygro. For generation of HMLE cells stably expressing Snail, the retroviral plasmid vector (pBabe puro Snail) was purchased from Addgene (#23347). Production of recombinant retroviruses using Plat-E packaging cells, and their infection and selection of the infected cells, were performed as described previously.^55^ During these experiments, we observed that expression of normal-p53 in shp53 cells using pLKO.1-based lentivirus or pcDNA-based plasmid caused cell senescence and death.

### Gene expression profiling

Cells were serum starved for 16 h, and then left untreated or treated with TGFβ1 for 2 h in the absence of FCS. Total cellular RNAs were then isolated using the RNeasy Mini Kit (Qiagen), according to the manufacturer's instructions. Analysis of the mRNA expression profiles was performed by Gene Chips (Yokohama, Japan), by use of the GeneChip Human GENOME G4851A arrays (Affymetrix, Santa Clara, CA, USA). The data normalizations were performed based on Gene Spring GX11 (Agilent Technologies, Santa Clara, CA, USA). Color visualization of the data was performed using Java TreeView software.

### Drug resistance

Gemcitabine, and 5-fluorouracil were purchased from Wako. Temsirolimus was purchased from Sigma-Aldrich. The stocks were prepared in dimethyl sulfoxide (Sigma-Aldrich) or PBS, aliquoted and stored at −20 °C, until use. Cells were plated into 96-well culture plates at 3000 cells per well, and drugs were applied on the next day. After being incubated for another 3 days, cell viabilities were measured. The experiments were repeated at least three times.

### Statistics

Unless otherwise noted, each *in vitro* experiment was performed in triplicate, and analysis of variance was used to compare two groups of independent samples (two-sided), assuming similar variance. Data are presented as mean±s.e.m. The log-rank test was used to compare Kaplan–Meier survival curves. *P*-values <0.05 were considered significant.
