# Supplementary material for: ZEB1 induces EPB41L5 in the cancer mesenchymal program that drives ARF6-based invasion, metastasis and drug resistance
Source: Oncogenesis. 2016 Sep 12;5(9):e259–. doi: 10.1038/oncsis.2016.60 (PMC5047961; doi:10.1038/oncsis.2016.60)
Supplement: Supplementary Information [file oncsis201660x1.doc]

**SUPPLEMENTARY INFORMATION**

**FOR**

**ZEB1 induces EPB41L5 in the cancer mesenchymal program that drives ARF6-based invasion, metastasis, and drug resistance**

**Ari Hashimoto^1^, Shigeru Hashimoto^1^, Hirokazu Sugino, Ayumu Yoshikawa, Yasuhito Onodera, Haruka Handa, Tsukasa Oikawa and Hisataka Sabe^*^**

Department of Molecular Biology, Hokkaido University Graduate School of Medicine, Sapporo 060-8638, Japan

^1^These authors contributed equally to this work.

**^*^**Correspondence: sabeh@med.hokudai.ac.jp

**Running Title: ZEB1-EPB41L5 axis driving breast cancer malignancy**

**Keywords: AMAP1, ARF6, drug resistance, EPB41L5, mesenchymal malignancy**

**SUPPLEMENTARY FIGURE LEGENDS**

**Supplementary Figure S1.** Silencing of EPB41L5 inhibits invasion and metastasis of MDA-MB-231 cells.

**(a**,**b)** EPB41L5 protein expression (**a**) and cell viability (**b**) of MDA-MB-231 cells, treated with siRNA oligonucleotides specific to *EPB41L5* or with an oligonucleotide bearing an irrelevant sequence (Irr). Protein levels were analyzed by immunoblotting using an anti-EPB41L5 antibody. β-actin was included as a control in (**a**). Cell viability was measured by the MTS assay. The results represent mean ± s.e.m. (*n* = 3), in which the viability of cells treated with Irr was normalized to 1.0 (**b**). (**c,d**) Silencing of *EPB41L5* in MDA-MB-231 cells. In **c**, EPB41L5 protein levels were analyzed by immunoblotting in luciferase-expressing MDA-MB-231 cells, transfected with shRNA plasmids for *EPB41L5* or with a scramble vector, in which a β-actin immunoblotting was included as a control. In **d**, Matrigel invasion activities of these cells were measured for 16 h in the presence of TGFβ1, as shown in Figure 1h. The results represent means ± s.e.m. of experiments performed in triplicate. ***P* < 0.01. (**e**) Time course of bioluminescence intensities emitted from the chests of each injected mouse. Five mice were analyzed for each group. Results were normalized as shown in Figure 1i. (**f**) Proliferation of cells, as indicated, was measured for 3 d *in vitro*. Relative cell growth was calculated by normalizing the values obtained from the Day 0 cells as 1. Error bars show means ± s.e.m. (*n* = 3).

**Supplementary Figure S2.** ZEB1 is responsible for EPB41L5 expression.

(**a**) Putative binding sites of ZEB1, found in the MatInspector promoter analysis tool, are shadowed. (**b**) *EPB41L5* mRNA levels in MDA-MB-231 cells, treated with siRNAs for *ZEB1* (1 and 2) or Irr. (**c**) Expression levels of *EPB41L5* and *ZEB1* mRNAs in HMLE cells, incubated with (+) or without (-) TGFβ1 for 12 d, and in HMLE-SNAI1 cells. MDA-MB-231 cells were included as a positive control. NC, without cellular mRNAs.

**Supplementary Figure S3.** p53 status does not affect cell cycle progression, cell viability, and Twist1/2 expression of MDA-MB-231 cells.

(**a,b**) Cell cycle distribution (**a**), as analyzed using BrdU and flow cytometry, and viabilities (**b**) of MDA-MB-231 cells (parental) and their p53 derivatives are shown. The results represent means ± s.e.m, in which the viability of the parental cells was normalized as 1.0 (*n* = 3). (**c**) Undetectable expression of *TWIST1* and *TWIST2* in MDA-MB-231 cells and their p53 derivatives. Heat maps are as shown in Figure 3a.

**Supplementary Figure S4.** ZEB1 and AMAP1 in resistance to chemotherapeutic drugs on MDA-MB-231 and MDA-MB-435s cells.

(**a**-**f**) MDA-MB-231 and MDA-MB-435s cells, pretreated with siRNAs for *AMAP1*, *ZEB1*, or Irr, were incubated with indicated doses of gemcitabine (**a,b**), 5-fluorouracil (**c,d**), and temsirolimus (**e,f**) for 3 d, and their viabilities were then measured. (**g**) ZEB1 and AMAP1 protein levels in MDA-MB-231 and MDA-MB-435s cells, treated with siRNAs for *AMAP1,* *ZEB1* or Irr, were analyzed by immunoblotting, as indicated. A β-actin immunoblot was included as a control. (**h,i**) *In vitro* growth of siRNA-treated MDA-MB-231 cells (**h)** and MDA-MB-435s cells (**i**). Each experiment was performed in triplicate, and the results are shown as means ± s.e.m. (*n* = 3). ***P* < 0.01.

**Supplementary Figure S5.** Analyses of TCGA RNASeq dataset with regard to relations between the EMT-related gene expression and the overall survival of patients and frequency of breast cancer subtypes.

(**a**) Kaplan-Meier curves do not show an association between high expression of *CDH2, VIM, TWIST1, TWIST2, SNAI1,* or *ZEB1* and the overall survival of breast cancer patients (*n* = 970). The database was analyzed by including the top 33% of primary breast tumors regarding their levels of the indicated mRNAs as the high-expression group. *P*-values represent the results of the log-rank test. (**b**) Frequency of breast cancer subtypes within high expression of the *EPB41L5*, ARF6 pathway components (*RTKs/GEP100/ARF6/AMAP1/EPB41L5*), or with *TP53* missense mutations as shown in Figure 5h.
