# Supplementary material for: ZEB1 induces EPB41L5 in the cancer mesenchymal program that drives ARF6-based invasion, metastasis and drug resistance
Source: Oncogenesis. 2016 Sep 12;5(9):e259–. doi: 10.1038/oncsis.2016.60 (PMC5047961; doi:10.1038/oncsis.2016.60)
Supplement: Supplementary Figures [file oncsis201660x2.pdf]

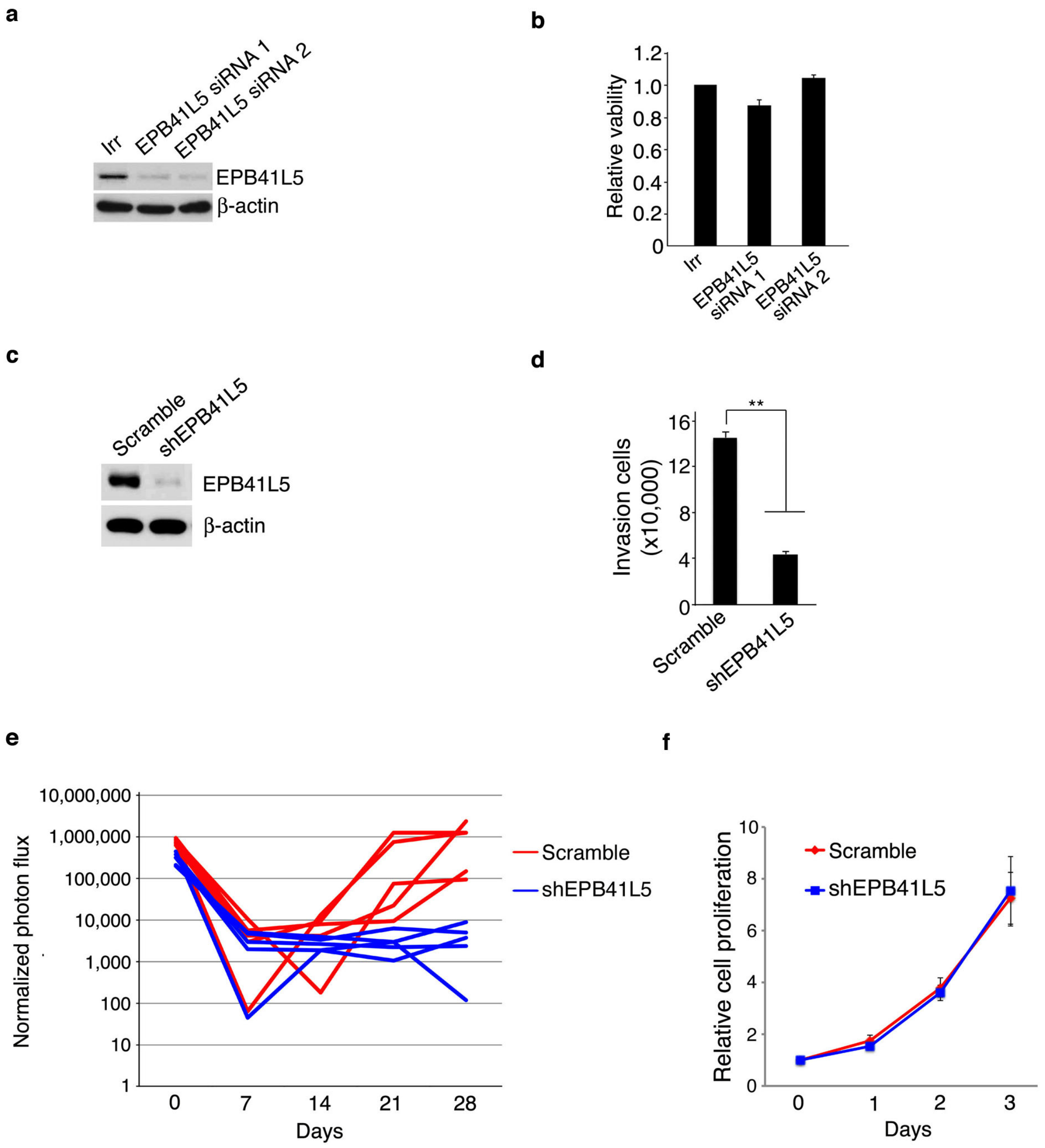

Supplementary Figure S1 Hashimoto et al

a

#1 -3950 TTTCTTCTCTGAATTAAAAGACAAGGCTTCAGGTAAAGGTCTTTTTCCCCTTCCCTTTTA -3891

#2 -2660 CTGGAGTGCAGTGCAGAGAAAGCAGCTCACTGAAACCTCCGCCTCCTGAATTCACGCGAT -2601

#3 -1550 GACCAGTGAAACCACCTTGGCAGCTCCAGCACCTGTTGAGCTGCCCAGCCAACACCACAT -1501

#4 -440 TAATCACCTTGTGACACCTATTTGTTTACTGTTTCCCCAGTAGAGTGAAAGTCACTTTAA -391

#5 -110 TAGTTCCAGCCCGGCCGGGCCCGGCCTCGCCGGTTTCTCTCCAGTCGCCGCGCCGGCCAA -51

b

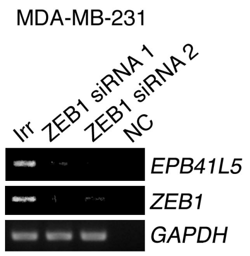

c

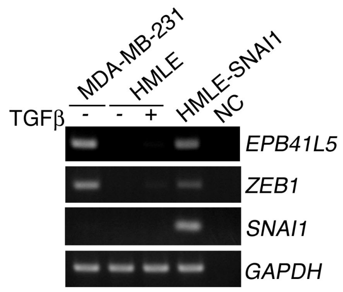

Supplementary Figure S2 Hashimoto et al

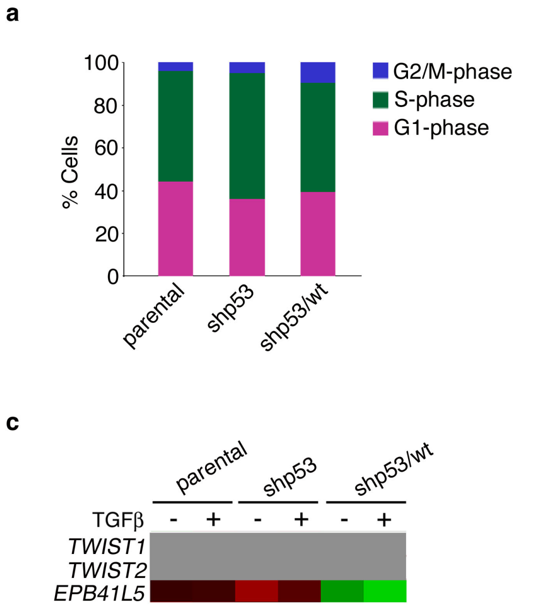

Supplementary Figure S3   Hashimoto et al

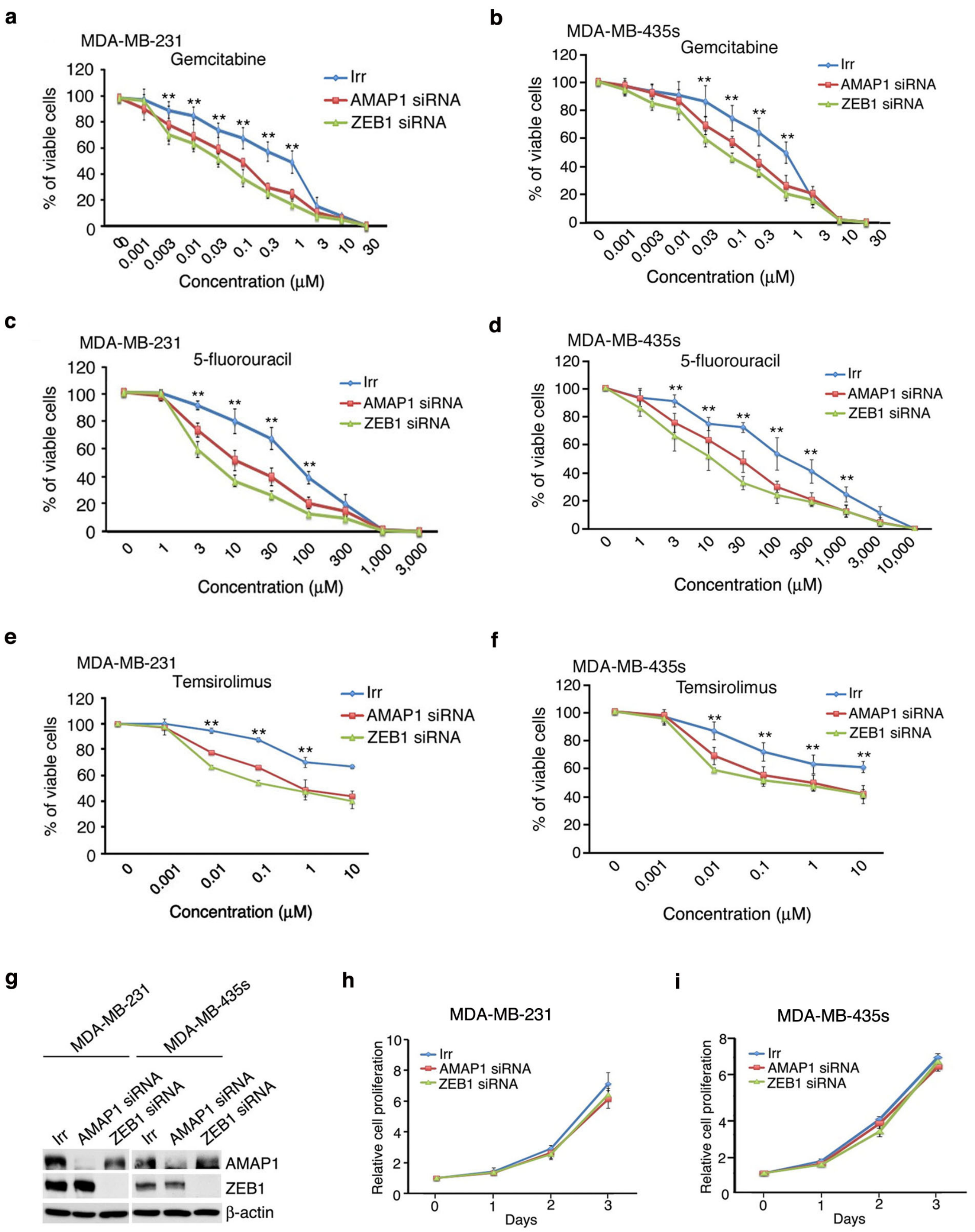

Supplementary Figure S4 Hashimoto et al

a

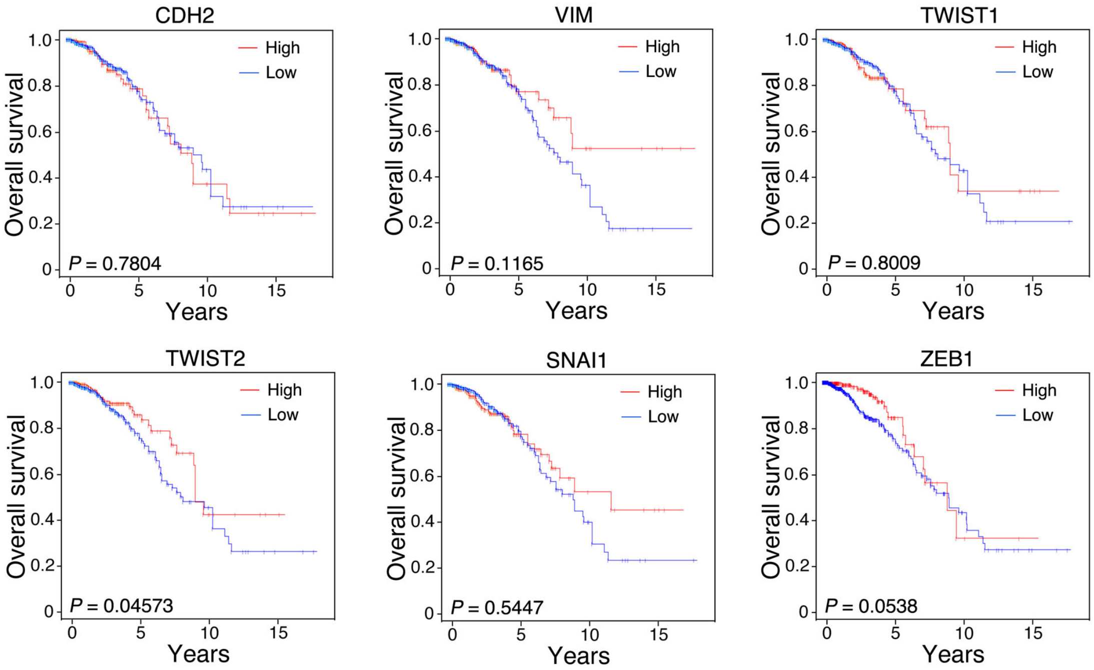

b

EPB41L5-high and missense TP53

Basal-like	1	(3.1 %)
HER2-enriched	2	(6.3 %)
Luminal A	6	(18.8 %)
Luminal B	8	(25.0 %)
ND	15	(46.9 %)

n = 32

EPB41L5-high

Basal-like	5	(1.5 %)
HER2-enriched	11	(3.4 %)
Luminal A	105	(32.5 %)
Luminal B	61	(18.9 %)
ND	141	(43.7 %)

n = 323

RTKs/GEP100/ARF6/AMAP1/EPB41L5-high

Basal-like	33	(10.5 %)
HER2-enriched	22	(7.0 %)
Luminal A	75	(24.0 %)
Luminal B	44	(14.1 %)
ND	139	(44.4 %)

n = 313

RTKs/GEP100/ARF6/AMAP1/EPB41L5-high and missense TP53

Basal-like	11	(19.3 %)
HER2-enriched	6	(10.5 %)
Luminal A	7	(12.3 %)
Luminal B	10	(17.5 %)
ND	23	(40.4 %)

n = 57

Supplementary Figure S5 Hashimoto et al
