# Supplementary material for: ZEB1 induces EPB41L5 in the cancer mesenchymal program that drives ARF6-based invasion, metastasis and drug resistance
Source: Oncogenesis. 2016 Sep 12;5(9):e259–. doi: 10.1038/oncsis.2016.60 (PMC5047961; doi:10.1038/oncsis.2016.60)
Supplement: Supplementary Tables [file oncsis201660x3.pdf]

**Table S1. Sequences targeted by siRNAs.**

| **Target Gene** | **Sequence (sense)** |
|---|---|
| Human *AMAP1* | AAGACCTGACAAAAGCCATTA |
| Human *EPB41L5* 1 | GAGAUGGAACUGGCUAUUUUU |
| Human *EPB41L5* 2 | UUCAGAUUCGUGCCUAUUCAG |
| Human *ZEB1* 1 | GACCAGAACAGUGUUCCAUGCUUAA |
| Human *ZEB1* 2 | GCUGAGAAGCCUGAGUCCUCUGUUU |
| Mouse *ZEB1* 1 | CCGGGUCAGUAAACAUACCUACAAA |
| Mouse *ZEB1* 2 | GACCAGAACAGUGUUCCAUGUUUAA |

## Table S2. Primers used for cloning of EPB41L5 and ChIP analysis.

### Cloning primers

| Name | Sequence (5'-3') |
|---|---|
| EPB41L5 N terminus Fw | AT GGATCC (*BamHI*) GCCACC (Kozak) ATGCTGAGTTTCTTCCGTAGAACA |
| EPB41L5 N terminus Rv | GA AGATCT (*BglII*) CATCCCCCAAGCCTGTTGGGA |
| EPB41L5 C terminus Fw | GA AGATCT (*BglII*) GCT CTG CCT GTG AGT CCT TCC |
| EPB41L5 C terminus Rv | AT GCGGCCGC (*NotI*) TCA (stop) GAGCTCAGTGGTCAGTAAACA |

### Primers for ChIP analysis

| Name | Sequence (5'-3') |
|---|---|
| #1 Fw | TTGGTCTGAGTTAGGGCAGGTAGTGTAGTT |
| #1 Rv | CTGCTTTGCCACCCTTAGCATGTTGACCT |
| #2 Fw | GAAGAAGGAATGCTTTTCGGGGTACCTGGC |
| #2 Rv | AGGCTCAAGAGTTCTAGCTCAGGAATTCGA |
| #3 Fw | TTGCCACACCAGCGGATCTGCAAGAGAGTA |
| #3 Rv | CTCATTGAGCTTCTTCTCGGGATTTGGCAC |
| #4 Fw | TCACCTTGTTAGAGTACCCTCCTGACCACA |
| #4 Rv | CGCAAATACTTATTGAGAGCCTGTCACG |
| #5 Fw | ACTGGGTTTCGGCAGCGCCCCTTTAAACCA |
| #5 Rv | GTAGACGCCGAGAAATGGCGCCCACAACGA |
